# Interaction Analysis of MRP1 with Anticancer Drugs Used in Ovarian Cancer: In Silico Approach

**DOI:** 10.3390/life12030383

**Published:** 2022-03-07

**Authors:** Absarul Haque, Ghazanfar Ali Baig, Abdulelah Saleh Alshawli, Khalid Hussain Wali Sait, Bilal Bin Hafeez, Manish Kumar Tripathi, Badrah Saeed Alghamdi, Hani S. H. Mohammed Ali, Mahmood Rasool

**Affiliations:** 1 King Fahd Medical Research Center, King Abdulaziz University, Jeddah 21589, Saudi Arabia; gbaig@stu.kau.edu.sa (G.A.B.); aalshawli@kau.edu.sa (A.S.A.); basalghamdi@kau.edu.sa (B.S.A.); 2 Department of Medical Laboratory Sciences, Faculty of Applied Medical Sciences, King Abdulaziz University, Jeddah 21589, Saudi Arabia; mrahmed1@kau.edu.sa; 3 Department of Biological Sciences, King Abdulaziz University, Jeddah 21589, Saudi Arabia; hmohammedali@kau.edu.sa; 4 Gynecology Oncology Unit, Obstetrics and Gynecology Department, Faculty of Medicine, King Abdulaziz University Hospital, Jeddah 21589, Saudi Arabia; khsait@kau.edu.sa; 5 Department of Immunology and Microbiology, South Texas Center of Excellence in Cancer Research, School of Medicine, University of Texas Rio Grande Valley, Edinburg, TX 78539, USA; bilal.hafeez@utrgv.edu (B.B.H.); manish.tripathi@utrgv.edu (M.K.T.); 6 Department of Physiology, Faculty of Medicine, King Abdulaziz University, Jeddah 21589, Saudi Arabia; 7 Center of Excellence in Genomic Medicine Research, Faculty of Applied Medical Sciences, King Abdulaziz University, Jeddah 21589, Saudi Arabia

**Keywords:** ovarian cancer, multidrug resistance, gemcitabine, carboplatin, multidrug resistance protein 1

## Abstract

Multidrug resistance (MDR) is one of the major therapeutic challenges that limits the efficacy of chemotherapeutic response resulting in poor prognosis of ovarian cancer (OC). The multidrug resistance protein 1 (MRP1) is a membrane-bound ABC transporter involved in cross resistance to many structurally and functionally diverse classes of anticancer drugs including doxorubicin, taxane, and platinum. In this study, we utilize homology modelling and molecular docking analysis to determine the binding affinity and the potential interaction sites of MRP1 with Carboplatin, Gemcitabine, Doxorubicin, Paclitaxel, and Topotecan. We used AutoDock Vina scores to compare the binding affinities of the anticancer drugs against MRP1. Our results depicted Carboplatin < Gemcitabine < Topotecan < Doxorubicin < Paclitaxel as the order of binding affinities. Paclitaxel has shown the highest binding affinity whereas Carboplatin displayed the lowest affinity to MRP1. Interestingly, our data showed that Carboplatin, Paclitaxel, and Topotecan bind specifically to Asn510 residue in the transmembrane domains 1 of the MRP1. Our results suggest that Carboplatin could be an appropriate therapeutic choice against MRP1 in OC as it couples weakly with Carboplatin. Further, our findings also recommend opting Carboplatin with Gemcitabine as a combinatorial chemotherapeutic approach to overcome MDR phenotype associated with recurrent OC.

## 1. Introduction

Ovarian cancer (OC) ranks fifth leading cause of death among women and one of the deadliest cancers among all female genital malignancies [1,2,3]. Despite the improvements and advancements in the diagnostic and therapeutic procedures, OC still remains the lowest survival disease among all gynecological malignancies in developed countries [2,4]. The high mortality rate of OC is due to the fact that 30% of the advanced stage tumors do not respond to standard chemotherapeutic regimens and the majority of the responders relapse over time [5,6]. Irrespective of the clinical stages, the current treatment modalities of OC include cytoreductive surgery followed by the first line of taxane and platinum-based chemotherapy to manage the cancer progression [7,8,9]. Despite the availability of a wide range of OC chemotherapeutic agents, none is fully effective in suppressing tumor growth due to the emergence of multidrug resistance (MDR) phenomenon. MDR is commonly attributed by its ability to efflux the therapeutic drugs out of the tumor cell by ABC transport proteins [10,11]. Often, MDR phenotype is treated by the use of Topotecan, Gemcitabine, Cisplatin, or Doxorubicin either alone or in a combination, depending on the type of MDR transporter involved in the given cancer [8,12]. Numerous findings have confirmed the involvement of ABC transporter, namely P-glycoprotein (P-gp), and the multidrug resistance associated protein (MRP) as major MDR pumps, and they are often responsible for chemotherapeutic failure in the OC [11,13]. P-gp and MRP transporters recognize similar substrates apart from few anticancer drugs such as taxanes (Paclitaxel and Docetaxel) which are preferable substrates for P-gp. Yet, both P-gp and MRP have been reported to be equally involved in conferring multidrug resistance in OC [14]. Despite the low structural and sequence homology between MRP1 and P-gp, yet they show overlapping substrate specificity such as binding to a wide range of substrates, and utilizing ATP hydrolysis to efflux the chemotherapeutic drugs out of cancer cells [15]. The MRP1, also known as ABCC1, has been reported to be actively engaged in translocating various chemotherapeutic agents in addition to diverse physiological substrates including glutathione conjugated drugs. Hence, it has been suggested to be playing a vital role in drug resistance phenotype in various cancers including OC [10,15]. The MRP1 transporter is a cell membrane channel with three transmembrane domains (TMD0, TMD1, and TMD2), and two cytosolic nucleotide-binding domains (N-terminal NBD1 and C-terminal NBD2) [16]. The TMDs are mainly involved in substrate identification and transportation whereas, the NBDs are responsible for ATP binding/hydrolysis, which is vital for energy-dependent efflux of MRP1 substrates [16,17]. It has been well documented that overexpression of MRP1 leads to efflux anticancer drugs and as a result MRP1 is considered as one of the major contributors of chemotherapeutic resistance in OC [18,19]. Of note, many mutations have been reported in the TMDs and NBDs of MRP1 that could affect the MRP1 protein folding and localization and hence, influence its substrate binding specificity which in turn could modulate chemotherapeutic response in OC patients [19,20,21] For example, a very common SNPs, G2168A, (arginine to glutamine) in MRP1 has been correlated with reduced drug transport activity and as a result increases the chemotherapeutic response in OC patients [20,22]. Therefore, it is highly interesting to investigate the MRP1 and its substrates interaction in order to determine the protein–ligand binding affinity and specificity with the wild-type and mutant protein transporter. To date, there is insufficient information available to address chemotherapeutic outcome with regard to the interaction of anticancer drugs with MRP1transporter. The MRP1 protein and its substrate specificity as well as its pharmacokinetics have been poorly understood by in silico analysis. Thus, understanding the MRP1 substrates specificities would have a major impact in designing novel chemotherapeutic drugs in order to minimize MRP1-related drug–drug interactions. In this present study, we aim to determine the binding site of MRP1 with various anticancer drugs using in silico analysis to determine the binding affinity and the potential interaction sites in full protein and to predict the best anticancer drugs that may be a promising choice to treat non-responder OC patients often caused due to overexpressed MRP1 transporter.

## 2. Materials and Methods

### 2.1. Ligand Preparation

**Figure 1:**
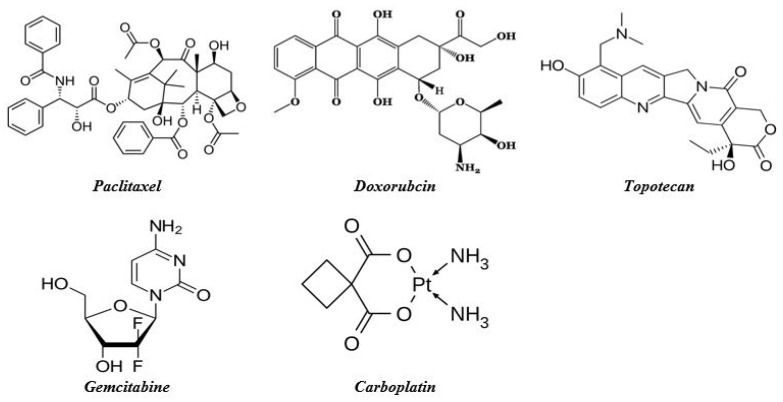
2D structure of anticancer drugs assessed in this study.

The 2-dimensional structures (2D) of the six drugs, Doxorubicin (PubChem ID: 31703), Gemcitabine (PubChem ID: 60750), Topotecan (PubChem ID: 60700), Paclitaxel (PubChem ID: 36314), and Carboplatin (PubChem ID: 426756) were retrieved from the NCBI PubChem database in .sdf format (https://pubchem.ncbi.nlm.nih.gov/, accessed on 18 December 2021) [22]. Whereas, the 2D structure of the ligands was converted into .mol2 format using Open Babel software as shown in Figure 1 [23].

### 2.2. Protein

Homology modelling of MRP1 was performed using *Bos taurus* MRP1 (PDB ID: 6UY0) as a template in Swiss-Model online software [24]. Sequence of Human MRP1 was retrieved from the UniPort database (Accession ID: P33527) [25]. The MRP1 protein structure was prepared for docking using BIOVIA discovery studio visualizer (Discovery Studio Visualizer v21.1.0.20298) [26]. The discrepancies in the structure (lacking hydrogens, removal of water molecules and ligands, orientation of the various functional groups) were examined and corrected.

### 2.3. Molecular Docking

Docking was performed by converting both receptor and ligands file to the .pdbqt format using AutoDock Tools (v1.5.6, https://autodock.scripps.edu, accessed on 19 December 2021). Molecular docking and calculation of binding affinity were performed using AutoDock Vina (https://vina.scripps.edu, accessed 19 December 2021) [27]. The results of molecular docking were visualized using a BIOVIA discovery studio visualizer [26].

## 3. Results

### 3.1. Homology Modelling of MRP1

**Figure 2:**
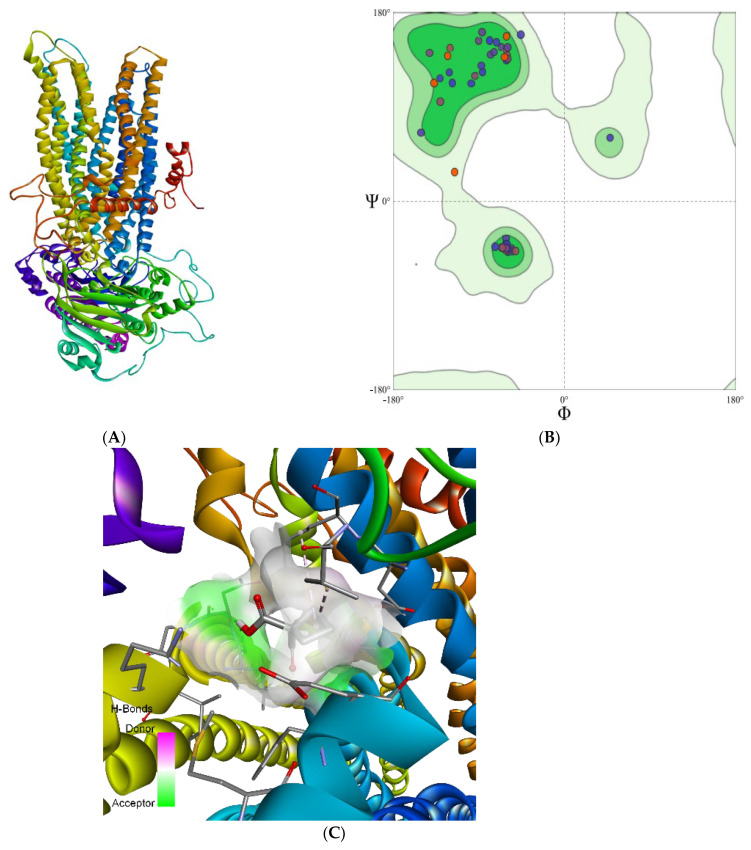
(**A**) Shows the predicted structure of MRP1; (**B**) Ramachandran map of MRP1; (**C**) pose of the docked complex in sphere indicates possible active site for ligand interaction.

Among the experimentally determined structures of ABC superfamily transport proteins, MRP1 of *Bos taurus* (PDD ID: 6UY0) was found to be the most appropriate template for human MRP1. Thus, the 3-dimensional structure of *Bos taurus* was used as a template to predict the structure of human MRP1 (Figure 2). The structure was predicted via Swiss Model and validated using Ramachandran plot analysis. Our results revealed that 95.70% of the residues were in the favored region where 0.98% were Ramachandran outliers (Figure 2). These values supported the structural validity of the predicted structure and were used for molecular docking analysis.

### 3.2. Analysis of Docking Results

The results of docking analysis relating to each drug are mentioned below. The binding affinities, binding sites, and number of hydrogen bonds are presented in Table 1.

#### 3.2.1. Paclitaxel Demonstrated Strong Interaction with MRP1

**Figure 3:**
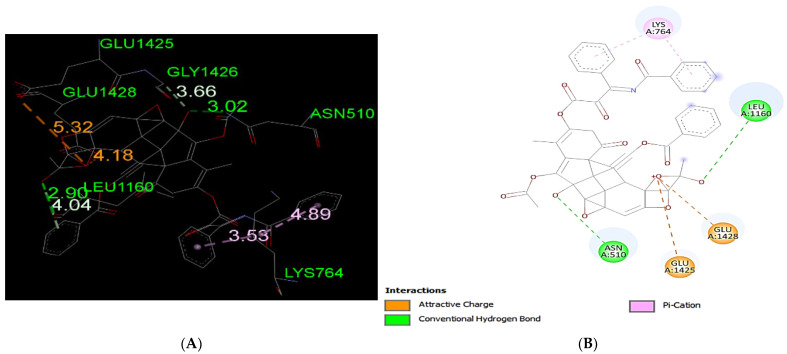
Predicted binding positions between MRP1 and Paclitaxel. (**A**) A structural view of the molecular docking; green dashed lines indicate hydrogen bonds, pink dashed lines represent pi–cation interactions, and the orange dashed lines represent attractive charge interactions. (**B**) Predicted Paclitaxel interactions; green dashed lines indicate hydrogen bonds, pink dashed lines represent pi–cation interactions, and the orange dashed lines represent attractive charge interactions.

The docking results of Paclitaxel with MRP1 (−11.3 kcal/mol) showed the lowest binding affinity, demonstrating the strongest interaction among all of the analyzed drugs (Table 1). The docking results revealed that Paclitaxel forms two hydrogen bond interactions with the MRP1 protein at each Asn510 and Leu1160 amino acid residue. The results also showed pi-cation interactions at residue Lys764 (Figure 3).

#### 3.2.2. Doxorubicin

**Figure 4:**
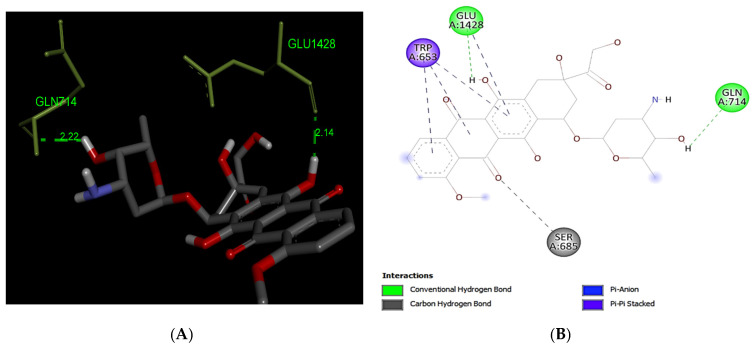
Predicted binding positions between MRP1 and Doxorubicin. (**A**) A structural view of the molecular docking; green dashed lines indicate hydrogen bonds. (**B**) Predicted doxorubicin interactions; green dashed lines represent the hydrogen bonds, purple dashed lines represent pi–pi stacking, blue dashed lines represent pi–anion interaction and gray dashed lines represents carbon hydrogen bond.

Our docking analyses of Doxorubicin has predicted the formation of two hydrogen bond interactions with MRP1 (−10.0 kcal/mol) at the residues Gln714 and Glu1428. Trp653 residue is predicted to form pi–pi stacked interaction with the aromatic ring of Doxorubicin, and it also formed pi–anion and carbon–hydrogen interaction between the ligand and MRP1 (Figure 4).

#### 3.2.3. Topotecan

**Figure 5:**
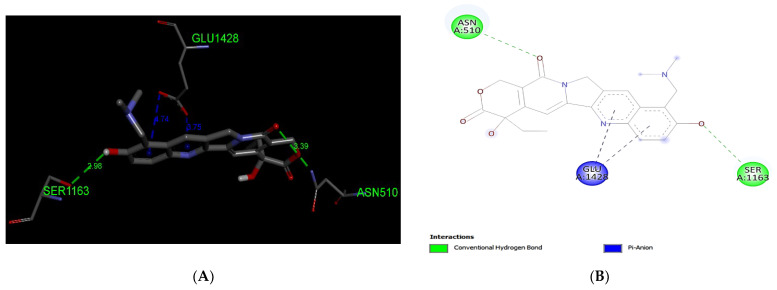
Predicted binding positions between MRP1 and Topotecan. (**A**) A structural view of the molecular docking; green dashed lines indicate hydrogen bonds, and blue dashed lines represent pi–anion interaction. (**B**) Predicted Topotecan interactions; green dashed lines indicate hydrogen bonds, and blue dashed lines represent pi–anion.

Our docking analysis of Topotecan has predicted the formation of two hydrogen bond interactions with MRP1 (−9.1 kcal/mol) at the residues Asn510 and Ser1163. Leu1160 residue is predicted to form pi–alkyl interaction with the aromatic ring of -Topotecan whereas the Glu1428 residue is predicted to form pi–anion interaction with MRP1 (Figure 5).

#### 3.2.4. Gemcitabine

**Figure 6:**
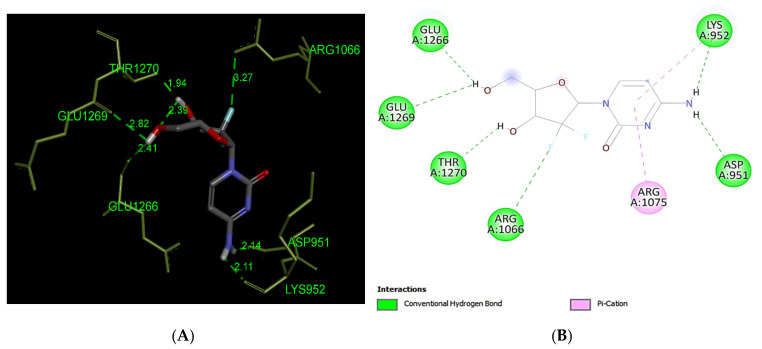
Predicted binding positions between MRP1 and Gemcitabine. (**A**) A structural view of the of molecular docking; green dashed lines indicate hydrogen bonds. (**B**) Predicted Gemcitabine interactions; green dashed lines represent hydrogen bonds, pink dashed lines indicate pi–cation interaction.

Our docking analysis of Gemcitabine has predicted the formation of two hydrogen bound interactions with MRP1 (−6.6 kcal/mol) at the residues Asp951, Lys952, Arg1066, Glu1266, Glu1269, and The1270 (Figure 6) whereas, the Lys952 Arg1075 residue forms pi–cation interaction.

#### 3.2.5. Carboplatin

**Figure 7:**
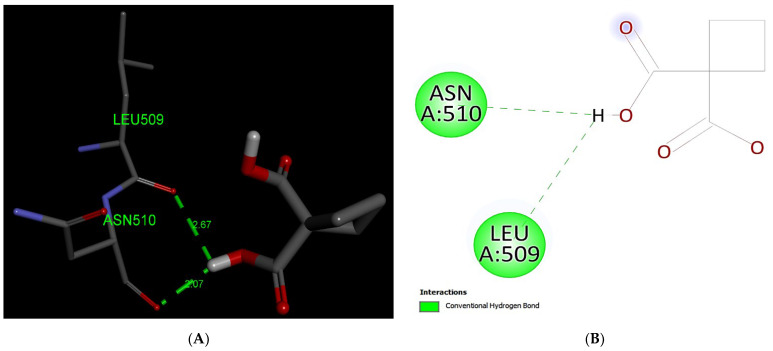
Predicted binding positions between MRP1 and Carboplatin. (**A**) A structural view of the molecular docking; green dashed lines indicate hydrogen bonds. (**B**) Predicted Carboplatin interactions; green dashed lines represent hydrogen bonds.

Our docking analysis of Carboplatin has predicted the formation of two hydrogen bonds with MRP1 protein at the residues Leu509 and Asn510 (Figure 7).

### 3.3. Residue Frequencies

Further, we confirmed our docking results using BIOVIA discovery studio visualizer which predicted that the residue Asn510 participated in the highest number of interactions with the analyzed drugs. Moreover, the maximum frequency of the residue interaction was observed with Paclitaxel, Topotecan, and Carboplatin.

### 3.4. Alignment of Ligands with MRP1 to Predict the Various Binding Sites/Position on Full Protein Structure

**Figure 8:**
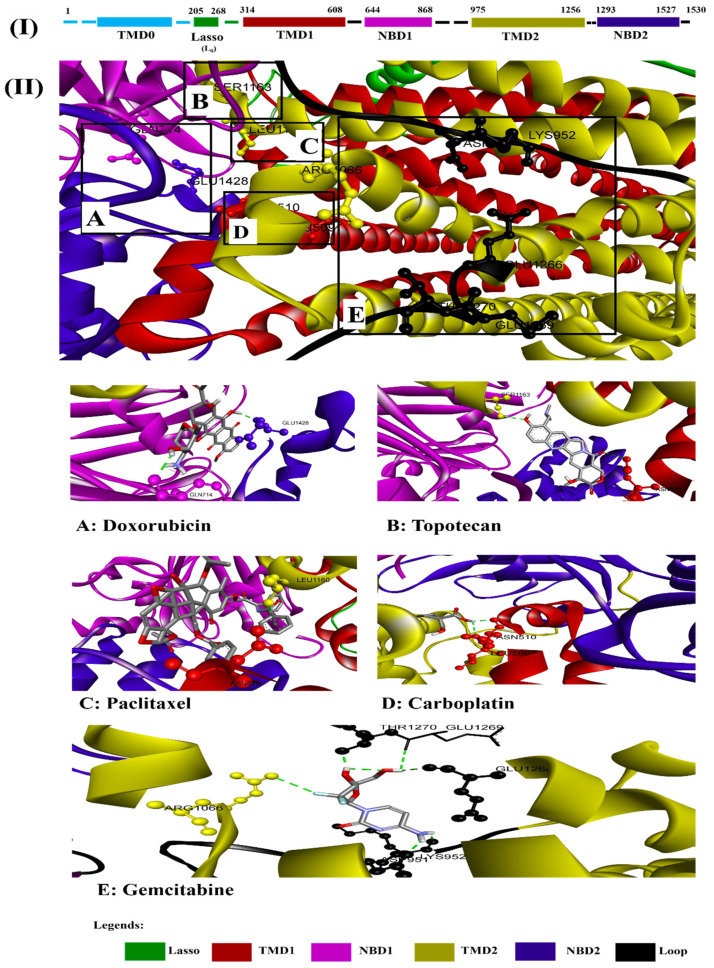
(**I**): Schematic diagram depicts topology of protein of MRP1. (**II**): Represents alignment of the structural position of MRP1 protein with respect to ligands binding sites depicting with different color; **II A** Shows the interaction of Doxorubicin with MRP1 in NBD1 (pink) and NBD2 (purple), **II** **B**,**C** illustrates the binding of Topotecan and Paclitaxel with MRP1 in TMD1 (red) and TMD2 (yellow) respectively, **II D** demonstrates the interaction of Carboplatin with MRP1 in TMD1 (red), and **II E** represents the binding sites of Gemcitabine with TMD2 (yellow) and loop (black) of MRP1.

We used BIOVIA discovery studio visualizer to align the different ligands with MRP1 in order to reveal the ligand binding sites and its spatial position at MRP1 protein, as shown in Figure 8. Our alignment comparison analysis shows that Paclitaxel and Topotecan bind TMD1 and TMD2 respectively, while Doxorubicin exclusively interacts with NBD1 and NBD2 of MRP1. However, Carboplatin binding site was found in TMD1 only. Interestingly, Gemcitabine was found to interact with six amino acid residues of MRP1 but notably, most of the binding residues (Asp951, Lys952, Glu1266, Glu1269, Thr1270) were located at loops of MRP1 except one residue (Arg1066) which was positioned at TMD2. Of note, Paclitaxel, Topotecan, and Carboplatin interact with a common amino acid residue (Asn510) in TMD1 of MRP1 protein.

## 4. Discussion

Platinum and taxane-based regimen is considered as the foremost choice for the treatment of OC patients [28]. However, multiple evidence show that the initial platinum-based chemotherapy appears to respond well to the early stages of OC, but prolonged exposure can lead to an increase in relapse cases. Thus advanced stages of tumor often fail to respond to the same line of treatment due to the emergence of MDR phenotype [28,29,30]. Notability, the development of MDR has been extensively associated with chemotherapeutic failure which consequently causes a rise in death rate to 90% in the advanced stages of OC patients [11]. Studies have demonstrated that the higher expression of MRP1 has a critical role in conferring drug resistance in OC [31,32]. In this perspective, MRP1 seems to be an ideal candidate to study with respect to ligand–protein interaction. Investigation of interaction between MRP1 and its substrate will shed some light on the binding affinity and specificity of protein-ligands in order to predict the least interacting drugs to MRP1 that may eventually serve as potential anticancer drugs to overcome the multidrug resistance in OC [32,33]. In the present study, we determined the binding site of MRP1 with various anti-cancers drugs using in silico methods to reveal the binding affinity as well as the potential interaction sites in MRP1. Our ligands–MRP1 protein alignment analysis revealed that Paclitaxel and Topotecan bind to TMD1 and TMD2 respectively. However, Carboplatin binding site was found in TMD1 only. Notably, Gemcitabine interacts with five amino acid residues (Asp951, Lys952, Glu1266, Glu1269, Thr1270) that lie in the loops of MRP1 except one amino acid (Arg1066) residue which binds to TMD2. In contrast, Doxorubicin binding position was observed exclusively at NBD1 and NBD2 of MRP1. Interestingly, Paclitaxel, Topotecan, and Carboplatin binding was involved with a common interacting amino acid residue (Asn510) that lies in TMD1 of MRP1 protein. The analysis of molecular docking revealed Paclitaxel has the highest binding affinity with MRP1 protein as shown in Table 1 and hence, signifies Paclitaxel as a preferable substrate of MRP1 Thus, Paclitaxel might not be considered as a suitable option for the treatment of MRP1-overexpressed OC patients. Paclitaxel is known as the most effective anti-cancer drug against OC, but its effectiveness reduced by the emergence of MDR phenotype often arise due to the overexpression of MRP1 in this cancer [34,35,36]. Remarkably, our findings also validate the above notions that Paclitaxel may not serve as a best choice in MRP1-overexpressed OC. On the other hand, Doxorubicin was found to be in second place in terms of the highest binding affinity after Paclitaxel with MRP1. Our docking analysis suggests that Doxorubicin is the most likely as a good substrate of MRP1 and hence it could not be considered as the best choice to treat overexpressed MRP1 OC patients. Our present observation further substantiates with the previous findings, where Doxorubicin was initially considered as a useful anticancer drug in many types of cancer especially, in breast and ovarian cancer but its wide use has been limited due to the growing concern of the reduced efficacy in MDR cancer background [37,38,39]. The docking analysis also showed that the binding affinity of Topotecan (−9.1 kcal/mol), Gemcitabine (−6.6 kcal/mol), and Carboplatin (−4.8 kcal/mol) ranked as third, fourth, and fifth, respectively as shown in Table 1. Interestingly, Topotecan and Carboplatin were found to be interacting with two amino acids each with the involvement of two hydrogen bonds as shown in Figure 5 and Figure 7, while the Gemcitabine having the second lowest binding affinities with MRP1 showed to interact with 6 amino acids residue with a total of 6 hydrogen bond formation as shown in Figure 6. Of note, the binding affinity of Topotecan was observed to be −9.1 kcal/mol which implies that Topotecan interacts weakly as compared to Paclitaxel (−11.3 kcal/mol) and Doxorubicin (−10.0 kcal/mol), thus it appears to serve as a better option for second line of chemotherapeutic drug as compared to Paclitaxel and Doxorubicin. However, Topotecan use has been limited due to MDR-associated drug resistance and toxicity [40]. Our findings matched with the previous observation where, Lee et al. 2020 have demonstrated that a combination of Topotecan with Cisplatin as a second line of palliative chemotherapy could be more effective in platinum-sensitive recurrent OC even though of having severe hematological and neutropenia toxicity [41].

Of note, our findings also illustrate that Gemcitabine and Carboplatin showed least binding affinity, which suggests that both drugs may poorly bind with MRP1 suggesting that both the anticancer drugs are not good substrates of MRP1. Hence, Gemcitabine and Carboplatin may serve as better chemotherapeutic options to overcome the MRP1-facilitated drug resistance. Our findings are also parallel with other studies where it has been suggested that Gemcitabine might be a potential treatment choice for platinum-resistant OC patients [42]. Interestingly, clinical studies carried out by Berg et al., 2019 and Lorusso et al., 2006 reported that Gemcitabine could be an effective and reliable chemotherapeutic drug for the treatment of platinum sensitive as well as resistant recurrent OC either alone or in combination with Carboplatin. Another study also conducted by Pfisterer et al. 2006 suggested that combination of Gemcitabine and Carboplatin considerably enhances progression-free survival and response rate without affecting quality of life for patients with platinum-sensitive recurrent OC [42,43,44]. Similarly, studies have proposed that a combinatorial approach of Gemcitabine along with platinum (Carboplatin or cis-platin) could be applied to treat platinum-resistant and recurrent OC which substantially improves the health of patients [44,45,46,47,48]. Further, combining Gemcitabine and platinum altogether have been suggested in breast cancer therapy in order to provide maximum clinical potential of these drugs. In parallel, our findings also suggested that Gemcitabine and platinum combination might be helpful to improve chemotherapeutic response to platinum-sensitive and relapse OC having MRP1 phenotype. Interestingly, alongside with conventional chemotherapeutic treatment, poly (ADP-ribose) polymerase (PARP) inhibitors also need to be considered in multidrug resistance OC patients in order to improve progression-free survival [3]. Thus, combination of platinum-based therapy and PARP inhibitors might be adopted as future treatment modalities in order to improve the efficacy of therapy especially in multidrug resistance OC patients. The chemotherapy resistance mechanisms have not been well studied in silico with regard to full protein of MRP1 and its substrates specificities in OC. Therefore, further research efforts are needed to identify the key ligand–protein (Chemotherapeutic drugs-MRP1) interactions with more anti-cancer drugs and MRP1 inhibitors.

The association between expression and functions of MRP1 with SNPs also needed to be considered in further studies as it may affect protein expression or function in order to get more insight toward determining high potency of such anticancer drugs. MRP1 gene polymorphism might be an indicator of the chemotherapeutic response in advanced OC. Thus, this approach certainly adds an extra leap toward exploiting and addressing selectivity of such chemotherapeutic agents to tackle drug resistance issues, as well as provides beneficial information pertaining to the discovery and design of novel and potent anticancer drugs that could possibly overwhelm MDR phenotype often associated with relapse cases of many cancers including OC.

## 5. Conclusions

In the current study, MRP1 was analyzed for docking against several anticancer drugs such as, Paclitaxel, Gemcitabine, Carboplatin, Doxorubicin, and Topotecan which are usually used to treat advanced stage ovarian cancer, to understand drug–target interactions. The 3D structure of MRP1 was modelled and docked to the drugs by using ADV. Our result showed that Paclitaxel has the highest binding affinity with MRP1, and it may be the substrate for MRP1. Carboplatin exhibits least binding affinity, which represents that it binds weakly with MRP1. Thus, Carboplatin appears to be more effective against MRP1. In conclusion, our findings suggest the possible use of Gemcitabine in combination with Carboplatin which may serve as a promising chemotherapeutic strategy for overcoming MDR in the relapse OC cases.

## Figures and Tables

**Table 1:** Docking and ligand binding affinity, binding sites, and number of H-bonds of MRP1 with anticancer drugs. *A.a (Amino acids).

Interaction of MRP1	Binding Affinity (kcal/mol)	*A.a Residues Forming H-Bond (s)
Paclitaxel	−11.3	2 (Asn510, Leu1160)
Doxorubicin	−10.0	2 (Gln714, Glu1428)
Topotecan	−9.1	2 (Asn510, Ser1163)
Gemcitabine	−6.6	6 (Asp951, Lys952, Arg1066, Glu1266, Glu1269, Thr1270)
Carboplatin	−4.8	2 (Leu509, Asn510)

## Data Availability

Not applicable.
